# A national-scale land cover reference dataset from local crowdsourcing initiatives in Indonesia

**DOI:** 10.1038/s41597-022-01689-5

**Published:** 2022-09-17

**Authors:** Hadi, Ping Yowargana, Muhammad Thoha Zulkarnain, Fathir Mohamad, Bunga K. Goib, Paul Hultera, Tobias Sturn, Mathias Karner, Martina Dürauer, Linda See, Steffen Fritz, Adis Hendriatna, Afi Nursafingi, Dian Nuraini Melati, F. V. Astrolabe Sian Prasetya, Ita Carolita, Kiswanto, Muhammad Iqbal Firdaus, Muhammad Rosidi, Florian Kraxner

**Affiliations:** 1 International Institute for Applied Systems Analysis (IIASA), Laxenburg, Austria; 2 World Agroforestry Centre, ICRAF Southeast Asia Regional Office, Bogor, Indonesia; 3 World Resources Institute Indonesia, Jakarta, Indonesia; 4 World Wildlife Fund (WWF) Indonesia, Jakarta, Indonesia; 5 Conservation & Development, Burung Indonesia, Bogor, Indonesia; 6 Agency for the Assessment and Application of Technology (BPPT), Jakarta, Indonesia; 7 Politeknik Pertanian Negeri Samarinda, Samarinda, Indonesia; 8 National Institute of Aeronautics and Space (LAPAN), Jakarta, Indonesia; 9 Forestry Faculty of Mulawarman University, Samarinda, Indonesia; 10 PT. Alam Bukit Tigapuluh, Jambi, Indonesia; 11 Yayasan Konservasi alam Nusantara, Jakarta, Indonesia

**Keywords:** Developing world, Geography, Environmental sciences, Sustainability

## Abstract

Here we present a geographically diverse, temporally consistent, and nationally relevant land cover (LC) reference dataset collected by visual interpretation of very high spatial resolution imagery, in a national-scale crowdsourcing campaign (targeting seven generic LC classes) and a series of expert workshops (targeting seventeen detailed LC classes) in Indonesia. The interpreters were citizen scientists (crowd/non-experts) and local LC visual interpretation experts from different regions in the country. We provide the raw LC reference dataset, as well as a quality-filtered dataset, along with the quality assessment indicators. We envisage that the dataset will be relevant for: (1) the LC mapping community (researchers and practitioners), i.e., as reference data for training machine learning algorithms and map accuracy assessment (with appropriate quality-filters applied), and (2) the citizen science community, i.e., as a sizable empirical dataset to investigate the potential and limitations of contributions from the crowd/non-experts, demonstrated for LC mapping in Indonesia for the first time to our knowledge, within the context of complementing traditional data collection by expert interpreters.

## Abstract (summary)

**Table:** 

Measurement(s)	land cover
Technology Type(s)	visual interpretation of satellite imagery
Sample Characteristic - Location	Indonesia

## Background & Summary

There has been considerable progress in automated, land cover (LC) mapping from national to global scales due to advances in the quality, variety, and accessibility of Earth Observation satellite data, along with improved capabilities in data processing using high-performance cloud computing and machine learning (ML) algorithms. Yet the availability and geographical diversity of LC reference data remains the main bottleneck to making substantial improvements to the accuracy of LC maps. The reference data refers to the labelled LC data which is considered the best available assessment of the ground condition^1,2^. The reference data is needed to train the ML algorithms and to assess the accuracy of the resulting LC maps. This is especially true in data scarce environments such as Indonesia, which is further compounded by the presence of heterogeneous landscapes and a diversity of land management practices.

The advent of the Internet opens up new possibilities for organizations to outsource human labelling tasks to the billions of internet users worldwide. Known as crowdsourcing^3^, this process of outsourcing work to the crowd is an attractive and promising approach to realizing a large, geographically-diverse LC reference dataset required for large-scale LC assessment^4–6^ such as at national scale in Indonesia. Additionally, crowdsourcing allows quicker and lower cost collection of such a large dataset than using professional surveying, which is important for spatially comprehensive LC monitoring. This also means that crowdsourcing opens up the opportunity for an open-source LC reference data repository, which will greatly benefit LC monitoring and assessment. For large-area mapping applications, using a large, but noisier, geographically representative set of labelled training data can result in a more accurate model^7^. This is when compared to using a smaller, more accurate, but geographically biased training dataset. Furthermore, such a citizen science approach, i.e., the public involvement in scientific research^8^, has the potential to be a sustainable data collection strategy such as that demonstrated by the OpenStreetMap initiative^9^, as well as raising awareness of LC issues and building up a citizen-based LC community.

The use of crowdsourcing for the collection of LC reference data requires two additional considerations: (i) the existing LC typology must be clearly defined and introduced as part of the data collection exercise to ensure high quality; and (ii) the prevailing socio-ecological context of the country in which the data are collected must be taken into account. The latter points to the fact that human-annotated data are never raw nor neutral, but instead reflect the pre-existing environmental context to which the annotators belong (in the case of LC data, the physical and social geography). However, the design of the LC scheme and LC class definitions are based on intended usage of the resulting LC map products. Therefore, discrepancy between operational concepts/definitions used by the stakeholders vis-à-vis technical considerations of the LC scheme/classification needs to be identified and addressed. This will ensure that the LC map products are relevant and can contribute effectively to the information needs of the national and sub-national stakeholders and end users of the LC products within the country. Hence, whenever possible, the LC labelling of the reference data should be undertaken by annotators who are familiar of the landscape (e.g., with experience on the ground), to capture valid perspectives, nuances, and contexts not immediately apparent to an observer with no local knowledge^10^. Furthermore, the efforts to maximize the involvement and active participation of local practitioners in generating the LC reference data may help to create a better understanding of the LC automated mapping system, and a greater sense of ownership of the downstream LC products. While LC visual interpretation has an element of subjectivity due to differences between human interpreters, gathering interpretations from those that represent the “interpretive community” may help to ensure that the agreement or disagreement among the interpreters can be generalized to those who have a stake in the data^11^.

Here we present LC reference datasets obtained from a national scale crowdsourcing initiative and a series of expert in-person workshops (or mapathons) in Indonesia, with an emphasis on local participation. These datasets are relevant for the LC mapping community, i.e., researchers and practitioners, as reference data for training ML algorithms and for map accuracy assessment (with appropriate quality-filters applied). The dataset is also useful for the citizen science community, i.e., as a sizable empirical dataset to investigate the potential and limitations of the crowd/non-experts, demonstrated for LC mapping in Indonesia for the first time to our knowledge, within the context of complementing traditional data collection by expert interpreters.

## Methods

**Fig. 1:**
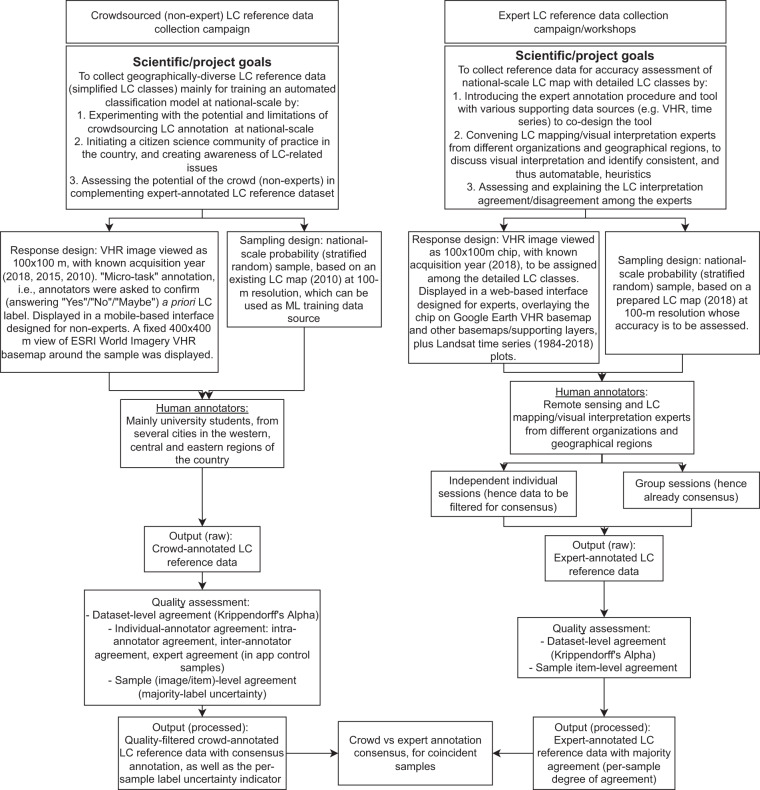
Schematic overview of the overall study and data collection design.

Two modes of data collection were designed, one for the crowd (i.e., the non-experts), and one for the experts, which was based on a consultation process with local experts in the country (Fig. 1). The designs considered Human-Computer Interaction trade-offs^12^ between annotation efficiency, annotation quality, agency of the annotators (i.e., user experience when performing the task), gamification elements^13^, and the engineering efforts required. For the crowdsourced annotation (performed by non-experts, or henceforth referred to as the crowd annotators), which was undertaken in a mobile application, the task was to accept or reject a previously assigned LC label based on a simplified seven class LC legend. Annotators were asked “Do you see <LC label> in more than half of the picture?”, which they answered with “Yes”,”No”, or”Maybe”.

In contrast, the expert annotation task (performed by local experts, or henceforth referred to as the expert annotators) was to select an LC label from a list of seventeen pre-defined LC classes from within a web application. Both the crowdsourced and expert annotations were based on interpretation of 100-by-100 m chips from very high spatial resolution (VHR) satellite images provided by the Digital Globe Viewing Service, where each chip was checked by multiple annotators to enable a consensus-based approach^2^ (or “crowd truth”^14^) in arriving at the final LC label with high confidence. Moreover, a whole-systems approach^15^ was adopted in which expert workshops (for expert annotators) and on site outreach events (for crowd annotators) were held to directly engage with the annotators to introduce the project, the scientific objectives of the data collection and the end-to-end data collection, processing, and dissemination process. Further details of the crowd annotation and expert annotation tasks are provided below.

### Crowdsourced LC reference data collection by non-experts (crowd annotations)

The main objective of the crowdsourced annotation was to generate reference data for training a supervised LC classification algorithm to produce a wall-to-wall map using satellite data at national scale, in a separate task in the project. A systematic sample of points spaced two km apart covering all of Indonesia was used to determine the availability of VHR imagery from the Digital Globe viewing service. From this, a stratified random sample (proportionally allocated by class area) was derived based on an existing, thematically detailed LC map for 2010. The sample allocation was additionally made proportional to the area of seven broad geographical regions (i.e., the main islands). The proportional allocation was based on stakeholder recommendations regarding the importance of having more reference data in LC classes with large areas. The total number of VHR image chips acquired was based on the available budget, which covered a range of years, i.e., 2018, for generating a more recent LC map; 2015, which was a year with an intense fire season to be examined as per the suggestion of a local stakeholder; and 2010, which may be used to improve the existing reference LC map.

The LC labels provided to the non-experts were based on the reference LC map from 2010. The original, detailed LC classes were simplified into the following seven generic LC classes: (1) Undisturbed Forest; (2) Logged Over Forest; (3) Oil Palm Monoculture; (4) Other Tree Based Systems; (5) Cropland; (6) Shrub; and (7) Grass or Savanna. This simplification was made to match the expected skills of the non-expert annotators, i.e., undergraduate students from any discipline at local universities in the country. To promote participation, preferably by those familiar with local landscapes, the local partners from the World Resources Institute (WRI) Indonesia, the World Agroforestry Centre (ICRAF) Southeast Asia Regional Office in Indonesia, and the World Wildlife Fund (WWF) Indonesia carried out outreach activities at sixteen Indonesian universities (10 universities in South Sumatra, 4 universities in East Kalimantan and 2 universities in West Papua) with students coming from many regions of the country. In the mobile app, the annotators could select the location in which the annotation tasks would be located from a set of broader geographical regions to align with their local knowledge if desired. In addition, they could select the LC class to be verified i.e., the image “pile”.

**Fig. 2:**
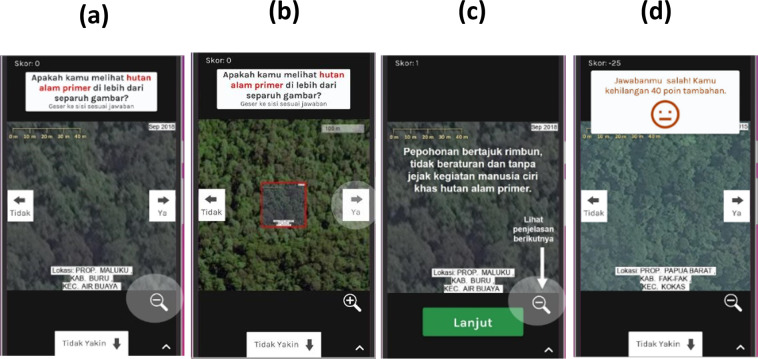
The crowdsourced non-expert LC annotation interface (in Indonesian) as a mobile application, made available at a dedicated local website (https://urundata.id/), which is based on the Picture Pile serious game available at https://geo-wiki.org/games/picturepile/. (**a**) The annotator was shown a VHR image chip (100-by-100 meters) with the question “*Do you see <prior LC label, e.g., ‘Undisturbed Forest’> in more than half of the picture*?”, which they then answered with “Yes” (swiping right), “No” (swiping left), or “Not Sure” (“Maybe”) (swiping down). The date of the image, the text stating the location (province, district, regency) of the sample, and a scale bar were shown. (**b**) Clicking the “zoom out” icon at the bottom right in (**a**) opens the image view of the larger-area (400-by-400 meters). (**c**) In the beginning the annotator went through example items with explanations. (**d**) When annotating a randomly shown control sample (image), the annotator received feedback regarding whether their answer was correct, and they were given a bonus point or a penalty accordingly.

In the mobile app (Fig. 2), the annotators were provided with a gallery of example image chips with the correct annotation and an explanation of why. To prevent poor quality annotations or insincere/malicious participation, a quality-control mechanism was implemented in the app during the crowdsourcing campaign. This was done by randomly showing control (or expertly-annotated) images during each sequence of ten images. The annotators then received feedback on whether their annotation agreed with the expert annotation; they were accordingly penalised or rewarded for their points as part of the gamification strategy. Efforts were made to ensure that the control images were reliable and representative of the variability of each LC type within each geographical region and of the answers (“Yes”, “No”, “Maybe”). One remote sensing expert from the country provided the annotation on the control samples with the help of reference LC maps as well as by consulting local experts whenever possible. In total, the number of unique control images represented 3.06% of the total number of images. However, in practice, the number of control images was greater than the number of unique items due to two reasons. Firstly, the same control item could be used as a “Yes” or “No” control item for different LC classes. Secondly, control items from different geographical regions were used together to minimize the repetition of control items. In practice, the control items per image pile in the mobile app were on average 29% of all images within the respective pile, with 19 out of 24 piles having more than 10% control images. To encourage participation, the campaign provided rewards, namely the opportunity to do a paid internship at either ICRAF Indonesia, WRI Indonesia, or WWF Indonesia, for the top three annotators in terms of total score (thus taking into account both quantity and quality of the annotations). A dedicated website in Indonesian was created (https://urundata.id/) to promote the crowdsourcing campaign and to help ensure sustainable engagement. Furthermore, the outreach campaign was held through offline seminars, webinars/workshops, and social media (i.e., Instagram @urundata and WhatsApp groups). Various channels were used for the dissemination to ensure that the campaign reached every target stakeholder (i.e., university students in city areas, university students in rural areas, researchers, and the general public).

The crowdsourced LC annotation campaign ran from 14 December 2019 to 28 April 2020, during which a total of 2,088,515 submissions were recorded in the mobile application. Around 10.6% of the annotations (i.e., 221,614) were control items while the remaining 1,866,901 annotations were for non-control items. When aggregated to majority per annotator per item, this corresponds to 928,139 unique annotator-unknown item pairs. The campaign recorded 145 days of activity (with 136 days having more than 100 annotations during the day).

**Fig. 3:**
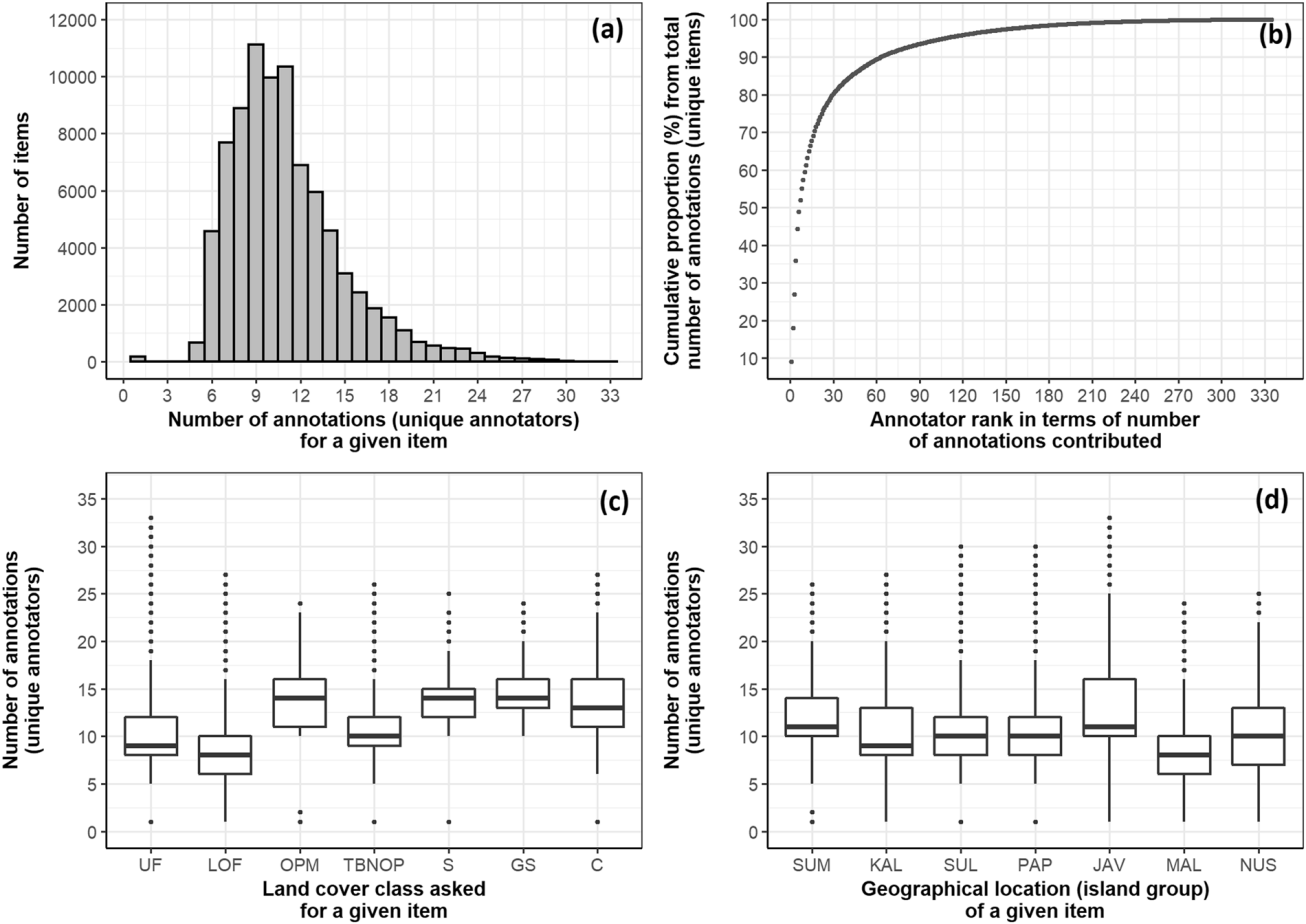
Descriptive plots describing the crowd-annotated LC reference dataset. (**a**) Distribution of the number of annotations (by unique annotators) across the items (samples, VHR image chips; excluding control items). (**b**) Distribution of the contributed annotations, for unique items, across the annotators, showing the contributions were more dominated by the top ranked annotators. (**c**) Distribution of the number of annotations (by unique annotators) across the prior LC class which the annotators were asked to accept/reject. UF: Undisturbed Forest; LOF: Logged Over Forest; OPM: Oil Palm Monoculture; TBNOP: Tree Based Not Oil Palm; S: Shrub; GS: Grass or Savanna; C: Cropland. (**d**) Distribution of the number of annotations (by unique annotators) across locations (geographical regions/major island groups) of the samples. SUM: Sumatera; KAL: Kalimantan; SUL: Sulawesi; PAP: Papua; JAV: Java, Madura, Bali; MAL: Maluku; NUS: Nusa Tenggara.

The mean number of annotation activities per day was 14,403, with a median of 9,631 and standard deviation of 15,279, ranging from 2 to 88,032 activities. The average time the annotator took to annotate an item was 2.4 seconds (standard deviation 10.4 seconds, median of all activities was 1 second). A total of 335 unique annotators registered and provided annotations, with the top 10 annotators providing around 60% of all annotations, while the top 5 annotators provided about 44% of all annotations (Fig. 3b). The mean number of unique annotators per day was 7, with a median and standard deviation of 5 and 9, respectively, ranging from 1 to 64 annotators. Overall, the median number of annotations per sample item was 10.0 annotations, with a median of 11.6 and standard deviation of 3.84, ranging from 1 to 33 annotations (Fig. 3a). The annotations appear well distributed across LC sample pixels belonging to different LC classes (Fig. 3c) and geographical regions (Fig. 3d).

### LC reference data collection by experts (expert annotations)

The main objective of the expert annotation activities was to generate reference data for performing an accuracy assessment of a wall-to-wall LC map produced using satellite data, in a separate task in the project. The LC map required a detailed legend of seventeen LC classes, and was developed for the year 2018. From the locations of available VHR image chips (see previous section), with the acquisition year 2018, a stratified random sample (proportional allocation) was derived based on the 2018 LC map as sampling strata. The detailed LC legend was designed together with local experts from ICRAF Indonesia for the purpose of a land restoration assessment at the national scale, taking into account the compatibility with existing classification schemes used in the country. Specifically, the detailed LC legend contains the following classes: (1) Undisturbed Dryland Forest; (2) Logged-Over Dryland Forest; (3) Undisturbed Mangrove Forest; (4) Logged-Over Mangrove Forest; (5) Undisturbed Swamp Forest; (6) Logged-Over Swamp Forest; (7) Agroforestry; (8) Plantation Forest; (9) Rubber Monoculture; (10) Oil Palm Monoculture; (11) Other Monoculture; (12) Grass or Savanna; (13) Shrub; (14) Cropland; (15) Settlement; (16) Cleared Land; and (17) Water Bodies.

**Fig. 4:**
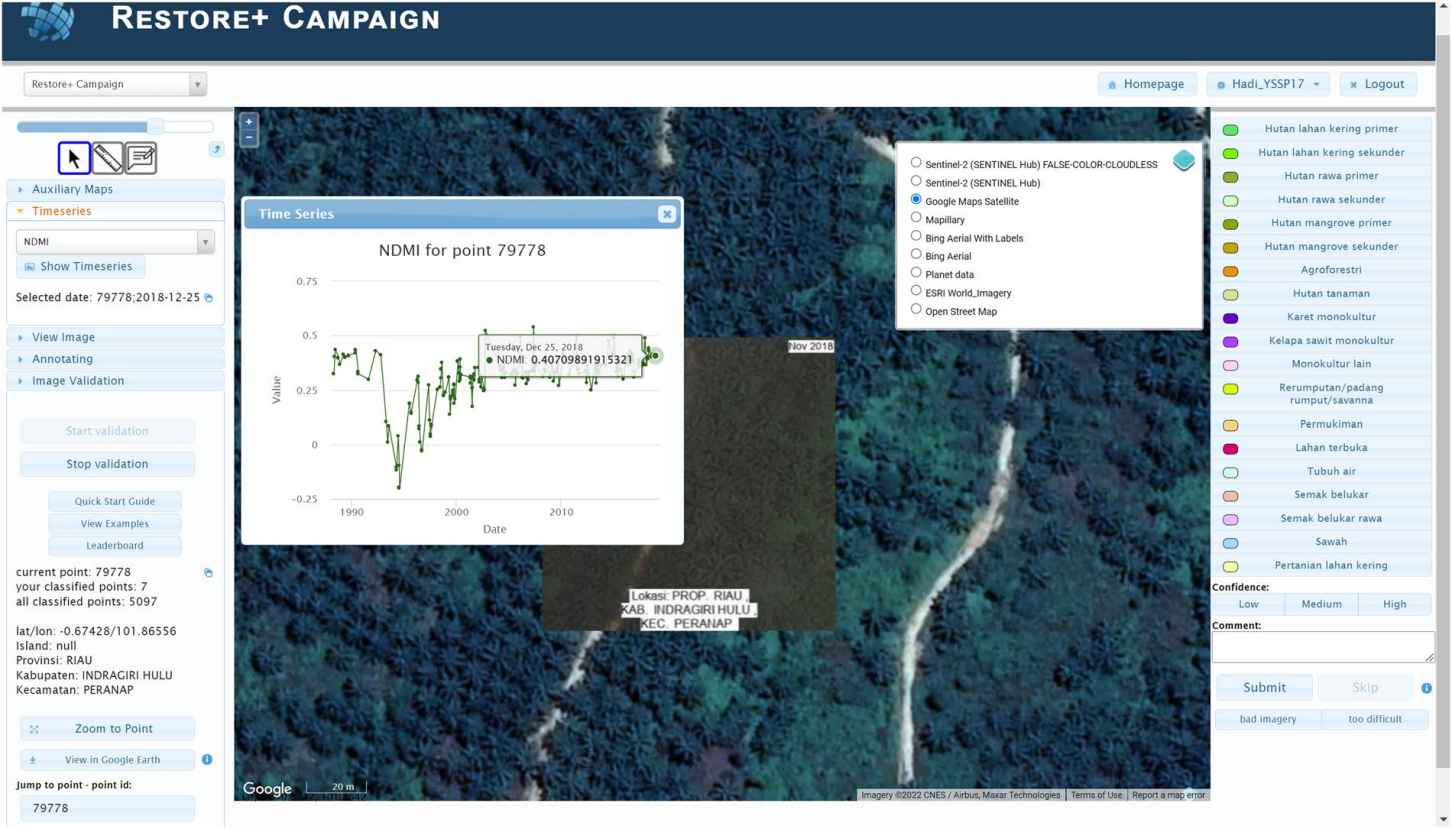
The expert LC annotation interface (in Indonesian) as a branch in the Geo-Wiki application. The Geo-Wiki application is available at https://www.geo-wiki.org/. A short guide to using the application, which describes the main features, is available at https://docs.google.com/document/d/1CcK4BleK7N-1EnoWZKD-tHq6h49ZBQKRrO16cUIcGv0/edit. Some particular features that the experts found useful were the ability to view and freely navigate the different VHR image basemaps, the Landsat time series and the corresponding Landsat image, the various auxiliary layers such as elevation, as well as the ability to measure distances, e.g., from roads.

**Fig. 5:**
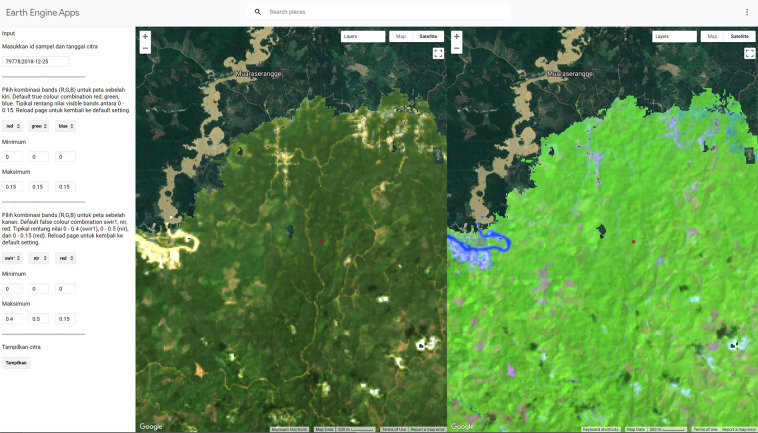
A companion Google Earth Engine (GEE) app (in Indonesian) to the expert LC annotation process using Geo-Wiki. The GEE app allows the experts to view Landsat images at the location of a selected sample for a selected Landsat observation date. The expert annotators can customize the display of the Landsat image. The app is available at https://hadicu06.users.earthengine.app/view/restoreplus-geowiki-companion.

Given the complex LC legend, the annotation task was designed for experts. Hence, the annotation interface provided additional support, e.g., other map layers, to determine the LC type. A dedicated branch of Geo-Wiki (Fig. 4), which is a visualization, crowdsourcing and validation tool for improving global land cover^16,17^, was developed, in which numerous crowdsourcing campaigns have taken place in the past^4–6^. The design of this branch was informed by a workshop with local experts. In particular, the application allowed the expert annotators to (1) freely zoom in and out (for landscape context) on the various VHR imagery basemaps (Google Maps Satellite, Microsoft Bing Aerial, ESRI World Imagery); (2) view various ancillary map layers, such as the Intact Forest Landscape layer^18^, a global mangrove map^19^, an elevation layer (SRTM^20^), ecoregions^21^, the Global Forest Change tree cover loss layer^22^, and two layers produced by from the Joint Research Centre of the European Commission: Global Surface Water layer^23^ and Global Human Settlement Layer^24^; (3) view Landsat historical time series (1984–2018) of various spectral indices; and (4) view Landsat images for selected dates in the companion app created using the Google Earth Engine Javascript API (Fig. 5). The last feature was added in consideration of the local interpreters, who are most familiar with using Landsat as primary data for visual interpretation of LC, especially in the development of the official land cover map by the MOEF^25^. The experts were instructed to base their LC labelling decision primarily on what is visible in the VHR image chip, which represents the sample pixel and the known image date.

Direct communications with the local experts were established to explain the end-to-end study design. In the end, the expert-annotated LC reference dataset was obtained from two kinds of interpretation session, namely in-person workshops (referred to as mapathons) and individual annotation sessions. The annotations made during the workshop already represent a consensus whereas the annotations made separately by the experts in the individual sessions needed to be postprocessed for consensus. In the individual sessions, to obtain LC labels with the highest confidence, a minimum of three annotations were required for each sample. The series of expert workshops brought together remote sensing and LC experts from government agencies (i.e., Ministry of Environment and Forestry; Forest Gazettement Agencies from Bandar Lampung, Yogyakarta, Makassar, Palu, Banjarbaru, Manokwari, Kupang, Tanjung Pinang, and Pekanbaru; National Institute of Aeronautics and Space (LAPAN); and Geospatial Information Agency (BIG); Agency for the Assessment and Application of Technology (BPPT)), civil society organizations (i.e., Burung Indonesia; FAO; TNC; Auriga; USAID IUWASH PLUS; Forest Carbon; and Wetlands International Indonesia), and universities (University of Indonesia; Universitas Indo Global Mandiri Palembang; Bogor Agricultural University; Mulawarman University; and Politeknik Pertanian Negeri Samarinda). The local experts were from the western, middle, and eastern regions of the country (with each of the major island regions of Sumatera, Java, Kalimantan, Sulawesi, and Papua represented). During the workshops, the expert annotators were divided into groups (Sumatera, Java-Madura-Bali, Kalimantan, Sulawesi-Maluku, and Papua-Nusa) to interpret the samples located in those geographical areas with which they had most familiarity. The workshops started with a discussion to build a common understanding, and thus consistent, transparent, objective, and reproducible interpretations^2^ of the LC legend and definitions, by going through selected examples together, including difficult edge cases (i.e., cases falling between two land cover classes). During the workshops, active discussion among the experts was encouraged to make explicit their interpretation process (perception and cognition, assumptions, visual cues, ground-based knowledge, etc.)^26^. As an incentive for active participation by the experts, in addition to sharing the collectively produced LC reference dataset, a training session on LC mapping using cloud computing in Google Earth Engine was provided during the workshop (recordings available in Indonesian at https://www.youtube.com/channel/UCY7fr6OtwumeIXDlWW9wd6A/videos). For the individual sessions, those experts who were able to annotate 500 samples were invited as co-authors on this publication.

The expert workshops were held on 12–13 February 2020 in Jakarta, Indonesia, followed by an online workshop on 10 June 2020. In the February workshop, nineteen local and regional LC experts were divided into seven interpreter groups based on familiarity with the geographical region. Each group of interpreters annotated between 15 and 77 samples (with an average among groups of 42). Each group of interpreters was accompanied by facilitators from ICRAF Indonesia. In the online workshop in June, 62 local and regional LC experts participated. During the LC annotation session, five interpreter groups were formed, with each group annotating between 30 and 82 samples (with an average of 46 across groups). The median length of time to annotate one item was 121 seconds in the February workshop, and 43 seconds in the online workshop in June.

The independent annotation activities were held between 10 June 2020 and 20 July 2020. Eleven LC experts actively participated, and by the end of the activities, eight experts had annotated around 500 samples each (who were then invited as co-authors on this paper) within 6.5 days (ranging from 3 to 9 days). The median time that the participating experts took to annotate one item was 41 seconds, which is similar to the group session in the online workshop. In the individual sessions, 1,450 samples had three annotations, 91 samples had two annotations, and 63 samples had one annotation.

From the expert LC reference data annotation activities, a total of 5,187 annotations was collected. Of these, 536 annotations were collected during expert workshops/mapathons and hence all 536 samples were already annotated with a consensus LC label, and 4,651 annotations covering 1,618 sample items were collected independently carried out using the web application.

### Post-hoc quality assessment measures for the human-annotated datasets

A known issue with crowdsourced data is the variable, and typically unknown, quality. In addition to the measures taken to prevent poor quality annotation during the annotation activities (such as providing guidelines, and using control samples for the crowd annotation), we implemented techniques for post-hoc detection of poor quality annotations^27^ as well as a quality assessment of the annotation data at the level of the entire dataset, individual annotators, and individual sample items. The use of several quality control measures, i.e., stability, reproducibility, and accuracy^28^, provides quantitative evidence for the reliability of the datasets. We note that data quality issues in crowdsourced data, and the choice of quality assessment metrics, are still an open area of research^29,30^. This is also true in the broader AI domain, i.e., currently there are no standardized metrics for characterising the goodness-of-data^31^. Research in the Volunteered Geographic Information (VGI) domain recommends the integration of several quality measures to produce more reliable quality information^1^. In our evaluation, we adopted the established practices on human annotation data quality assessment related to inter-rater reliability (inter-rater agreement) from content analysis and the related social science literature^12,28^. This is a practice that we encourage for further adoption by the LC mapping community as it can be expected that human-annotated labels will be increasingly collected to address training data bottlenecks in realizing the full potential of modern ML and AI algorithms. The fundamental property of quality assessment metrics is that they correct the observed agreement for expected chance agreements.

We first took the majority annotation made by an annotator for an image that they annotated more than once. If there was no majority, we kept the last annotation that the user made for that image. Dataset-level agreement was measured with the statistic called Krippendorff’s Alpha^28,32^. Krippendorff’s Alpha is a generalization of several known reliability indices, which has the benefits of being applicable to any number of annotators (not just two), any number of categories, and large and small sample sizes alike, as well as dealing with bias in disagreement, and is invariant to the selective participation of the annotators, i.e., it can deal with the fact that not every item is annotated by every annotator. The expected agreement is the data frequency. A Krippendorff’s Alpha value of 1.00 indicates perfect reliability while 0.00 indicates absence of reliability^28^.

Individual-annotator agreement was measured in terms of intra-annotator agreement (or stability), inter-annotator agreement (or majority agreement and reproducibility), and expert-agreement (or accuracy, estimated with control samples). These metrics were summarized by image pile to account for the potential variability in the task difficulty and the annotator’s skill with respect to the LC class or/and geographical region of the image. Intra-annotator agreement was calculated as the proportion of times the annotator’s annotation agreed with their previous annotation for that same image. The intra-annotator agreement values per annotator and per image were then averaged into per annotator and per pile. Expert agreement was calculated as the proportion of times the annotator correctly annotated the control samples. Expert agreement was calculated by image pile. The expected agreement was the data frequency^12^, i.e., the label frequency of the control items that appeared during the campaign. Inter-annotator agreement was calculated as the proportion of images on which the annotator agreed with the majority of annotations made by the other annotators for that same image. The expected agreement was the most frequent label of the control images that appeared in each pile during the campaign. The inter-annotator agreement values per annotator and per image were then averaged into per annotator and per pile. The three individual-annotator agreement metrics were considered together to assess the credibility of the annotator. The metrics were calculated per image pile in the crowd-annotated data, and thus, we account for potential variations in crowd skills for different LC classes or/and geographical regions.

### Determining the final most confident LC annotated label for each sample item

For the crowd-annotated dataset, to determine the final consensus annotation for each sample, we aggregated the annotations based on a weighted majority scheme with the credibility score of each annotator as weights. That is, the overall confidence of each possible annotation (“Yes”, “No”, or “Maybe”) for each sample was estimated as the sum of credibility scores (weights) of the annotators who provided each annotation respectively, divided by the sum of credibility scores of all annotators that annotated that sample. The annotation that had the highest value of overall confidence was determined as the final consensus annotation, with the uncertainty of that final consensus label estimated as one minus the overall confidence value (“least confidence” uncertainty sampling^12^). In our experiments, inferring the individual annotator’s credibility based on individual-annotator expert agreement alone, and excluding annotations from annotators with a negative credibility score, was found to provide the highest dataset accuracy as assessed with the best available gold-standard reference in this study, namely the expert-annotated data with the majority label. For the expert-annotated dataset, the final label for samples with a majority was used, i.e., for samples obtained from the group sessions and from independent individual sessions, samples with a percent majority of more than 50%.

## Data Records

All data are available on the figshare repository^33^. The Data Records comprise tabular data and are organized into five groups (Tables 1–5). All data are in comma-separated.csv format. We provide the raw (unprocessed) data as well as the processed data, i.e., filtered for consensus. The former would facilitate further analysis of the data while the latter would facilitate easier direct use of the data as reference data, e.g., for training ML models (see Usage Notes). No personal data are contained in the Data Records. Below we briefly describe the groups of files, but a detailed description of each table is provided in the Supplementary File 1.

**Table 1:** Summary of data files included in the dataset, Group 1: SAMPLE_METADATA.

File name	Description	Rows	Columns
locations.csv	List of unique locations (coordinates of the centroids) of the sample pixels (i.e., VHR image chips).	70418	3
images.csv	List of unique sample pixels (i.e., VHR image chips).	83949	5
samplesLocation.csv	List of sample pixels and their coordinates.	83949	2
locationsRegion.csv	List of unique locations and the broad geographic regions they fall into.	70418	2
samplesPile.csv	List of sample pixels and the image group (“pile”) they belong to in the mobile application for crowd annotation. The “pile” corresponds to an LC type and a broad geographical region.	83949	2
piles.csv	List of the image groups (“piles”) in the mobile application for crowd annotation.	24	3

See header information of the tables in Supplementary File 1.

**Table 2:** Summary of data files included in the dataset, Group 2: CROWD_ANNOTATIONS.

File name	Description	Rows	Columns
crowdAnnotationsRaw.csv	LC annotations (accepting/rejecting a priori LC label) made by the crowd (using the mobile application), unprocessed.	1866901	2
crowdAnnotationsRawInfo.csv	Further information about the annotations made by the crowd, unprocessed.	1866901	4
crowdAnnotationsPerAnnotatorMajority_.csv	Annotations made by the crowd, summarized to a unique record (majority) per annotator, per image.	928139	3
crowdAnnotationsConsensusPerSample_.csv	Annotations made by the crowd, processed to obtain a consensus/majority annotation per image.	69800	4
crowdAnnotators_expertAgreement_.csv	Agreement of the crowd annotators with expert annotation on the in-app control images. Summarized per annotator, per pile.	872	6
crowdAnnotators_intraAnnotatorAgreement_.csv	Intra-annotator agreement of the crowd annotators. Summarized per annotator, per pile.	498	6
crowdAnnotators_interAnnotatorAgreement_.csv	Inter-annotator agreement of the crowd annotators. Summarized per annotator, per pile.	896	7
crowdAnnotatorsSummaryScorePerSamplePerLabel_.csv	Summary of the crowd annotations and their credibility scores, per sample, per answer.	83943	11
crowdAnnotatorsSummaryScorePerSamplePerLabel_annotatorsFiltered_.csv	Summary of the crowd annotations and their credibility scores, per sample, per answer, with annotations from low-performing annotators excluded.	76514	11

The files with suffix “_” in the file names are processed annotation data. See header information of the tables in Supplementary File 1.

**Table 3:** Summary of data files included in the dataset, Group 3: EXPERTS_ANNOTATIONS.

File name	Description	Rows	Columns
expertsAnnotationsRaw.csv	LC annotations (selecting an LC label to be assigned to the sample) made by the local experts (using the web application), unprocessed.	5187	4
expertsAnnotationsRawInfo.csv	Further information about the annotations made by the local experts, unprocessed.	5187	6
expertsCommentsTrans.csv	Free-text comments entered by the local experts, in Indonesian and the English translation.	285	2
expertsAnnotationsConsensus_.csv	Annotations made by the local experts, processed to obtain a consensus/majority annotation per sample.	1715	3

The files with suffix “_” in the file names are processed annotation data. See header information of the tables in Supplementary File 1.

**Table 4:** Summary of data files included in the dataset, Group 4: CROWD_CONSENSUS_VS_EXPERTS_CONSENSUS_ANNOTATIONS.

File name	Description	Rows	Columns
crowdConsensusVsExpertsConsensusAnnotations_.csv	Samples with both consensus annotation from the crowd, and consensus annotation from the local experts.	1298	3

The files with suffix “_” in the file names are processed annotation data. See header information of the tables in Supplementary File 1.

**Table 5:** Summary of data files included in the dataset, Group 5: AUXILIARY_FILES.

File name	Description	Rows	Columns
landCoverClassesConversion.csv	Correspondence between LC classes in the simplified scheme (for crowd annotation) and the detailed scheme (for annotation by local experts).	17	2
Table S22	Definition of the land cover classes as provided by the local experts.	—	—

See header information of the tables in Supplementary File 1.

Group 1 (Table 1, header information in Tables S1–S6) contains metadata information about the sample units, i.e., the LC sample pixels and the corresponding VHR image chips. Of note is that the samples were derived from a single reference map, and for the same location, there might be several images (i.e., VHR image chips) corresponding to different image acquisition years. In the crowdsourced annotation, the images were grouped into “piles” in the annotation interface, with the piles corresponding to the LC class and the geographical region of the sample location (see files “samplesPile.csv” (header information in Table S5) and “piles.csv” (header information in Table S6)). A link to the data in Group 5 (Table 5) can be made to obtain the LC class name and definition.

Group 2 (Table 2, header information in Tables S7–S15) contains the crowd-annotated LC reference dataset. The files “crowdAnnotationsRaw.csv” (header information in Table S7) and “crowdAnnotationsRawInfo.csv” (header information in Table S8) together contain the unprocessed annotation data, i.e., as submitted by the non-expert interpreters in the mobile application. The most important file is “crowdAnnotationsConsensusPerSample_.csv” (header information in Table S10), which is the processed annotation data containing the final, most confident, consensus annotation for each sample, with the estimated uncertainty (as described in Methods). The other files contain the individual-annotator agreement metrics (observed agreement, expected agreement, and chance-adjusted agreement), calculated per image pile (see Group 1), which are only required if data users want to apply custom data filtering based on their specific use case (see Usage Notes). Link the data to Group 1 for information about the samples such as the geographic locations.

Group 3 (Table 3, header information in Tables S16 to Tables S19) contains the expert-annotated LC reference dataset. The files “expertsAnnotationsRaw.csv” (header information in Table S16) and “expertsAnnotationsRawInfo.csv” (header information in Table S17) together contain the unprocessed annotation data, i.e., as submitted by the expert annotators in the web application. The file “expertsAnnotationsConsensus_.csv” (header information in Table S19) contains the processed annotation data, i.e., samples with among-experts majority/consensus label. The type or degree of consensus reached for each sample was stored. Refer to Group 5 for the description of the detailed LC legend used in the expert annotation. A link to the data in Group 1 can be made to obtain information about the samples, such as the geographic locations.

Group 4 (Table 4, header information in Table S20) contains coincident samples between the crowd-annotated dataset and the expert-annotated dataset with the consensus label. It was based on files in Group 2 and Group 3. The expert labels were converted into binary labels to align with the crowdsourcing task, and they were used as a gold-standard set for accuracy assessment of the crowd-annotated dataset. A link to the data in Group 1 can be made to obtain information about the samples, such as the geographic locations.

Group 5 (Table 5, header information in Table S21; Table S22) covers the auxiliary files describing the LC classes as provided by the local experts, of which two classification schemes i.e., a simplified and a detailed scheme, were used for the crowd annotation and expert annotations, respectively. Files in other groups refer to this group for LC class information.

**Fig. 6:**
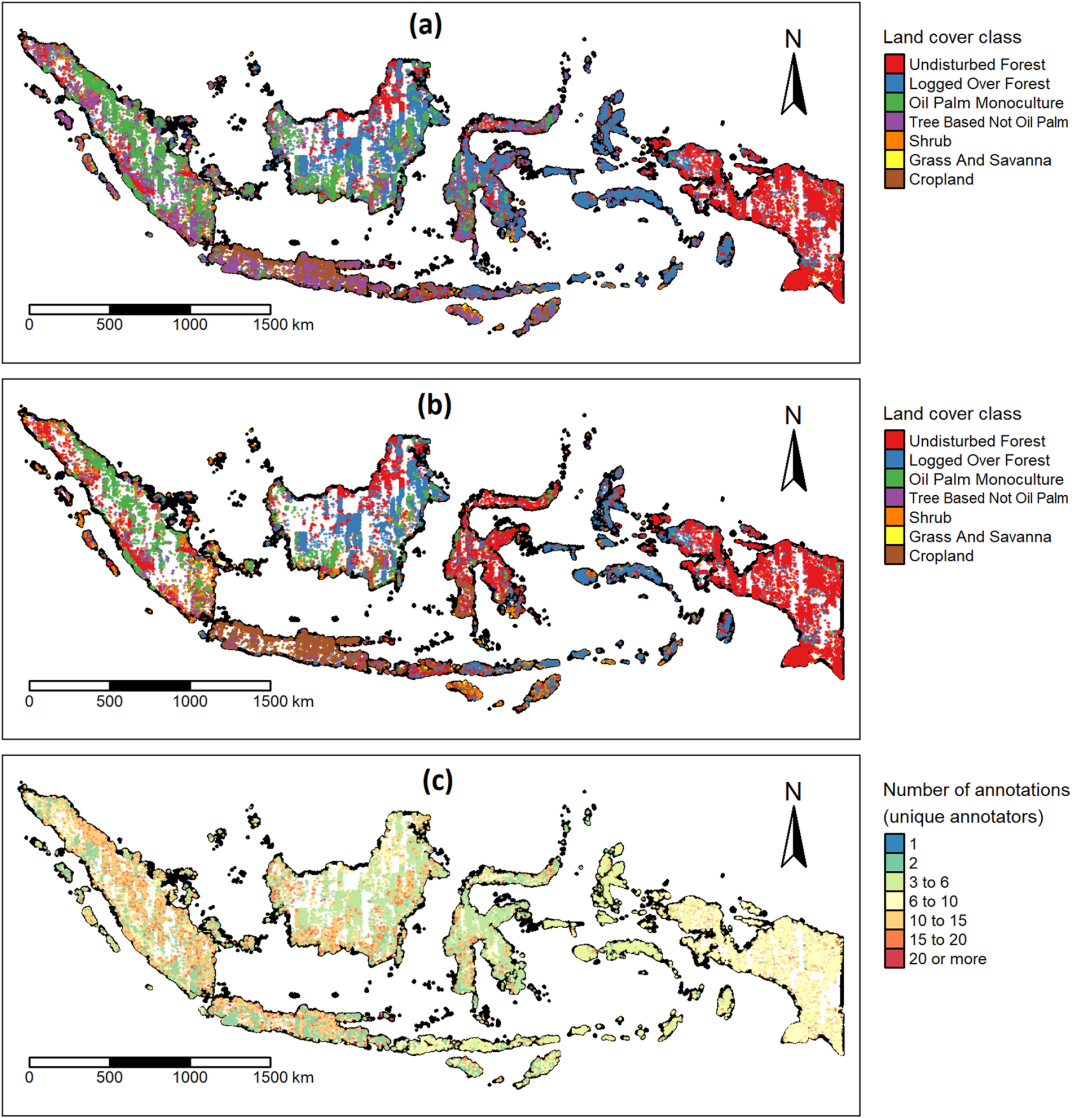
Locations, annotations, and the number of annotations of the crowd-annotated LC reference data. (**a**) All samples (thus crowd consensus/majority answer can be “Yes” or “No”), coloured by the LC label asked to be accepted/rejected in the annotation task (simplified LC legend). (**b**) Samples with crowd consensus/majority answer “Yes” (thus confirming the prior LC label) and number of annotations (by unique annotators) of at least two. (**c**) Number of annotations by unique annotators (excluding control items). Note in all (**a**–**c**): (i) if an annotator made multiple annotations for a sample (item, VHR image chip), the majority annotation from that annotator for that sample was used; (ii) annotations from annotators with expert-agreement scores worse than chance were filtered out.

The quality-filtered crowdsourced annotation data (i.e., with annotations from low-performing annotators excluded) shows a good spatial distribution across the whole country (Fig. 6). The number of annotations (by unique annotators) was generally more than three at all locations, with a good spread of locations having up to more than ten annotations particularly in Sumatera, Java, and Kalimantan (Fig. 6c). Samples with consensus “Yes” annotation, and thus known LC label and hence usable as training data for supervised classification, also appear well distributed across the country, as well as within each major island (Fig. 6b). Notably, Tree Based Not Oil Palm samples were generally lacking in the dataset after quality filtering (Fig. 6b), which is due to the low expert agreement scores (i.e., with annotations excluded from the annotators with an expert agreement score worse than chance) of the individual annotators when annotating this LC class (see data file “crowdAnnotators_expertAgreement_.csv” (header information in Table S11)).

## Technical Validation

**Fig. 7:**
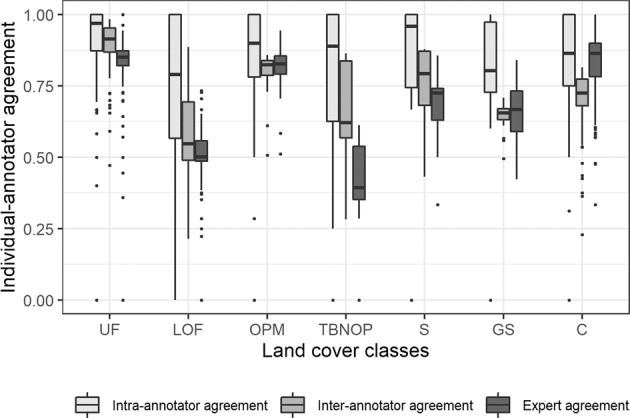
Three measures of the reliability of the annotators: intra-annotator agreement or stability (consistency), inter-annotator agreement or reproducibility, and agreement with experts for the sample items belonging to the different LC classes. Note the actual (observed) agreement values are shown here. If an annotator made multiple annotations for a sample item, the majority annotation from that annotator for that sample was used. UF: Undisturbed Forest; LOF: Logged Over Forest; OPM: Oil Palm Monoculture; TBNOP: Tree Based Not Oil Palm; S: Shrub; GS: Grass or Savanna; C: Cropland.

We consider three types of indicator to demonstrate that the datasets produced by the crowd are reliable, i.e., high intra-annotator agreement (i.e., consistency), high inter-annotator agreement (i.e., consensus) and good agreement of the crowdsourced data with the expert data. In the crowd-annotated dataset, the three individual-annotator agreement statistics are high for Undisturbed Forest, Oil Palm Monoculture, and Cropland (Fig. 7) but low for Logged Over Forest and Tree Based Not Oil Palm, suggesting caution is needed in using samples belonging to these two classes without further verification. Among the three metrics, intra-annotator agreement for all LC classes is on average higher than the rest, suggesting that the annotators are generally consistent in their labelling. Moreover, lower agreement with the experts tends to correspond to lower inter-annotator agreement. We note, however, that the accuracy of the crowd is more reliably assessed with consensus labels from the local experts, which would allow more generalizable conclusions to be made about the accuracy of the crowdsourced dataset.

The dataset-level agreement of the crowd-annotated dataset, which was a measure of the reliability of the whole dataset in terms of reproducibility, and which is the most feasible kind of reliability to test for^28^, was found to be high in terms of the observed agreement (Table 6). However, when adjusted for the expected agreement by chance, the agreement (Krippendorff’s Alpha) was generally low, with the exception of Oil Palm Monoculture and Cropland classes, which have moderate agreement (probably because these two classes are the easiest to visually identify from VHR imagery). We note, however, that the chance-adjusted agreement values were likely underestimated due to the high expected agreement, which was in turn due to the “prevalence” problem that is known to cause agreement statistics to be unrepresentatively low^34^. The prevalence problem is expected here given the nature of the annotation task, i.e., to accept/verify a prior LC classification with “Yes”, “No” or “Maybe”. Thus, the frequency of the “Yes” response is expected to be much higher, the “No” response is much lower and the “Maybe” response was found to be rare (<1% of all submitted annotations). In this situation, both the expected and chance-corrected agreement need to be considered to appropriately interpret the agreement statistics^34^.

**Table 6:** Dataset-level agreement of the crowd-annotated LC reference dataset. Images annotated by only one unique annotator are excluded.

Cases	Number of samples	Observed agreement	Expected agreement	Krippendorff’s Alpha
Samples belonging to all classes prior label	83769	0.78	0.67	0.34
Samples belonging to “Undisturbed Forest” prior label	24360	0.93	0.92	0.12
Samples belonging to “Logged Over Forest” prior label	18002	0.65	0.63	0.05
Samples belonging to “Oil Palm Monoculture” prior label	5495	0.81	0.48	0.63
Samples belonging to “Tree Based Not Oil Palm” prior label	17236	0.74	0.73	0.03
Samples belonging to “Shrub” prior label	4730	0.79	0.79	0.02
Samples belonging to “Grass or Savanna” prior label	1679	0.65	0.54	0.23
Samples belonging to “Cropland” prior label	12267	0.72	0.51	0.42
Samples with VHR image chips in RGB	47141	0.79	0.68	0.34
Samples with VHR image chips in grayscale	36628	0.78	0.66	0.34
Samples with VHR image chips acquired in 2010 (larger background image was not displayed)	21234	0.77	0.65	0.34
Samples with VHR image chips acquired in 2015 or 2018	62535	0.79	0.68	0.34
**Annotations made by annotators with expert agreement worse than expected chance agreement are excluded**	76425	**0.80**	0.63	**0.45**
Annotations made by annotators with inter-annotator agreement (majority agreement) worse than expected chance agreement are excluded	58916	0.73	0.60	0.33
Annotations made by annotators with either expert agreement or inter-annotator agreement (majority agreement) worse than expected chance agreement are excluded	51345	0.71	0.51	0.41

See Table 3 for number of samples with consensus response “Yes”, and thus the LC label is known, i.e., the prior LC label is accepted/verified.

Comparing the overlapping samples from the expert annotated dataset with the crowd majority annotations showed moderate agreement (Table 7). We note, however, that the assessment samples here are limited and are thus not readily generalizable to the whole population of items in the crowd-annotated dataset; further assessment with more gold-standard reference samples is warranted. For example, there are no overlapping samples for Cropland and Oil Palm Monoculture classes, but we have already shown relatively high dataset-level agreement for these classes (Table 6).

**Table 7:** Agreement between majority annotation of the crowd and experts.

Land cover	Crowd majority annotation (task was to verify if the image shows the given land cover)	Number of crowd-annotated samples	Number of available coincident experts-annotated samples with expert majority (consensus) annotation	Simple agreement (%) between crowd majority annotation and expert majority annotation
Undisturbed Forest	All	24360	449	55
	Yes	24283	446	54.7
	No	77	3	100
	Maybe	0	—	—
Logged Over Forest	All	17871	438	49.8
	Yes	8905	214	58.4
	No	8828	224	41.5
	Maybe	138	—	—
Tree Based Not Oil Palm	All	3405	80	62.5
	Yes	1202	21	28.6
	No	2161	59	74.6
	Maybe	42	—	—
Shrub	All	4730	90	31.1
	Yes	4 715	89	30.3
	No	15	1	100
	Maybe	0	—	—
Grass or Savanna	All	1679	40	55
	Yes	1137	22	18.2
	No	542	18	100
	Maybe	0	—	—
Oil Palm Monoculture	All	5488	NA	NA
	Yes	3091	NA	NA
	No	2259	NA	NA
	Maybe	138	—	—
Cropland	All	12267	NA	NA
	Yes	6420	NA	NA
	No	5840	NA	NA
	Maybe	7	—	—

The expert LC label was reclassified into the crowd annotation classes, and then converted into “Yes” and “No”. The gold-standard assessment samples are expert-annotated samples with consensus (majority) annotation. NA denotes that coincident expert-annotated samples are not available.

Notably, despite the high individual-annotator agreement metrics for the “Undisturbed Forest” class (Fig. 7), the comparison with experts was not high (Table 7). This can be partly explained by the fact that two different interfaces were used, i.e., the online Geo-Wiki by the experts, which has much more information compared to the mobile app used by crowd. To infer whether a forest is undisturbed or logged, signs of human activity need to be seen, often within a large radius from the sample location. This can be further augmented by knowledge of the legal status of the forest estate, which the experts could access but is not available in the mobile app. Due to this, caution is needed in using the crowd-annotated dataset for Undisturbed Forest and Logged Over Forest classes. Without further information, it is recommended to combine these into a single class of Forest.

## Usage Notes

These datasets offer an evaluation of the potential and limitations of involving the general public (i.e., non-experts) in large-scale LC monitoring initiatives, which for the first time is demonstrated in Indonesia. In this regard, the expert-annotated LC reference dataset (some samples with comments from the expert annotators) offers a unique opportunity to investigate the pattern and underlying causes of disagreement among-experts (which has been very rarely documented^2^), and to progressively build community consensus in a bottom-up manner, rather than the possibly less effective approach of imposing rigid interpretation rules in a top-down manner. It is important to note that in some cases, disagreement can probably only be resolved by incorporating objective (unambiguous) evidence from an on-the-ground perspective. The samples of both experts and the crowd can be used to understand the intrinsic differences (i.e., perception and cognition) between the crowd and experts^35^ in LC visual annotation tasks, and in turn, help to improve future follow-up crowdsourcing initiatives, e.g., in terms of providing better instructions/guidelines to the crowd.

We provide the raw and the quality-filtered crowd-annotated dataset along with data quality control measures. The latter is particularly useful in allowing users to extract an optimal subset of the crowdsourced data for their particular use case. Concretely, the users may choose the samples items based on the label uncertainty score provided to find the threshold that yields the desired accuracy as evaluated against a gold-standard reference sample^12^. Additionally, the data users may use different ways to assign credibility scores to the annotators based on the individual-annotator agreement statistics also provided in the dataset, or/and exclude annotations from annotators judged as inadequately skilled, based on their credibility scores (Table 7).

The datasets provided can be used as training data for a supervised LC classification model using satellite data to produce an LC map. The large-scale coverage, and the large sample size of the LC reference data provided here, help to prevent shifts in the distribution of features and labels between the training data and the domain where the model is applied, and thus allows for the development of a more robust and transferable LC classification model^36^. For this use case of the crowdsourced data (that inevitably contain some amount of label errors), it is, instead, more useful to optimize the classifier quality, instead of the data quality (i.e., quality of the reference data itself). That is, data users can perform end-to-end supervised classification experiments, and filter the crowd-annotated data to be used as training data, based on the impact (e.g., of varying uncertainty thresholds used in the consensus/majority label) on the downstream predictive skills of the classification (ML) model. Used as training data, there is typically a trade-off between quality (degree of noise in the labels) and quantity (as well as class allocation/balance, and geographical diversity) of the examples^7^. Modern ML algorithms may be robust to some degree of noise in the training data, especially if the training data are of a large size, and thus covers the variations in features and labels with respect to the population. Data users may also experiment with ML algorithms that explicitly account for the uncertainty in the training data (label noise)^37^ as indicated by, e.g., the uncertainty of the final (consensus/majority) label in the crowd-annotated dataset.

The dataset provided here may also be used as evaluation data for ML models, or statistically robust map accuracy assessment. However, additional expert review^2^ of the relevant subset of the data is strongly recommended to eliminate any potential label errors. The large number of samples also makes it possible to create a more spatially continuous accuracy assessment of the LC map^38^, or to have the assessment performed covering different sub-national geographical extents. Finally, the dataset provided here can contribute towards the continuous improvement of regional and global LC maps^39^.

## Supplementary information

Supplementary Information

## Data Availability

All data and code are available without restrictions from figshare^33^.
