# Supplementary material for: A national-scale land cover reference dataset from local crowdsourcing initiatives in Indonesia
Source: Sci Data. 2022 Sep 17;9:574. doi: 10.1038/s41597-022-01689-5 (PMC9482649; doi:10.1038/s41597-022-01689-5)
Supplement: Supplementary file 1 — Supplementary Information [file 41597_2022_1689_MOESM1_ESM.docx]

**Supplementary Information**

### A national-scale land cover reference dataset from local crowdsourcing initiatives in Indonesia

Hadi, Ping Yowargana, Muhammad Thoha Zulkarnain, Fathir Mohamad, Bunga K. Goib, Hultera, Tobias Sturn, Mathias Karner, Martina Dürauer, Linda See, Steffen Fritz, Adis Hendriatna, Afi Nursafingi, Dian Nuraini Melati, F.V. Astrolabe Sian Prasetya, Ita Carolita, Kiswanto, Muhammad Iqbal Firdaus, Muhammad Rosidi, Florian Kraxner

Corresponding author: Hadi (hadicu06@gmail.com)

**This PDF file includes:**

Tables S1 to S22.

**Table of contents:**

Table S1. Header of *locations.csv*.

Table S2. Header of images.csv.

Table S3. Header of samplesLocation.csv.

Table S4. Header of locationsRegion.csv.

Table S5. Header of samplesPile.csv.

Table S6. Header of piles.csv.

Table S7. Header of crowdAnnotationsRaw.csv.

Table S8. Header of crowdAnnotationsRawInfo.csv.

Table S9. Header of crowdAnnotationsPerAnnotatorMajority_.csv.

Table S10. Head of crowdAnnotationsConsensusPerSample_.csv.

Table S11. Header of crowdAnnotators_expertAgreement_.csv**.**

Table S12. Header of crowdAnnotators_intraAnnotatorAgreement_.csv.

Table S13. Header of crowdAnnotators_interAnnotatorAgreement_.csv.

Table S14. Header of crowdAnnotatorsSummaryScorePerSamplePerLabel_.csv.

Table S15. Header of crowdAnnotatorsSummaryScorePerSamplePerLabel_annotatorsFiltered_.csv.

Table S16. Header of expertsAnnotationsRaw.csv.

Table S17. Header of expertsAnnotationsRawInfo.csv.

Table S18. Header of expertsCommentsTrans.csv.

Table S19. Header of expertsAnnotationsConsensus_.csv.

Table S20. Header of crowdConsensusVsExpertsConsensusAnnotations_.csv.

Table S21. Header of landCoverClassesConversion.csv.

Table S22. Definition of the land cover classes as provided by the local experts.

**Group 1: SAMPLE_METADATA**

Table S1. Header of locations.csv.

| **Variable** | **Explanation** |
| --- | --- |
| *LocationID* | Unique number identifying each location of the sample pixel (i.e., VHR image chip). |
| Lat | Latitude of a location centroid (decimal degrees). |
| Lon | Longitude of a location centroid (decimal degrees). |

Table S2. Header of images.csv.

| **Variable** | **Explanation** |
| --- | --- |
| *SampleID* | Unique number identifying each sample pixel (i.e., VHR image chip). |
| ImageYear | Acquisition year of the image chip (2018, 2015, 2010). |
| ImageMonth | Acquisition month of the image chip (1-12). |
| ImageDay | Acquisition day of the image chip (1-31). |
| ImageColourType | Colour type of the image chip (1=RGB, 2=panchromatic). |

Table S3. Header of samplesLocation.csv.

| **Variable** | **Explanation** |
| --- | --- |
| *SampleID* | Unique number identifying each sample pixel (i.e., VHR image chip). Note that more than one image chip (i.e., with different acquisition years) can correspond to one locationID. |
| LocationID | Unique number identifying each location of the sample pixel (i.e., VHR image chip). |

Table S4. Header of locationsRegion.csv.

| **Variable** | **Explanation** |
| --- | --- |
| *LocationID* | Unique number identifying each location of the sample pixel (i.e., VHR image chip). |
| Region | Unique number identifying the geographical region in which the sample pixel is located (1=Sumatera, 2=Kalimantan, 3=Sulawesi, 4=Papua, 5=Java Madura Bali, 6=Maluku, 7=Nusa Tenggara). |

Table S5. Header of samplesPile.csv.

| **Variable** | **Explanation** |
| --- | --- |
| *SampleID* | Unique number identifying each sample pixel (i.e., VHR image chip). |
| PileID | Unique number identifying the pile (i.e., group of items) of which the sample pixel (i.e., VHR image chip) belongs to in the crowd annotation interface. |

Table S6. Header of piles.csv.

| **Variable** | **Explanation** |
| --- | --- |
| *PileID* | Unique number identifying the pile (i.e., group of items) of which the sample pixel (i.e., VHR image chip) belongs to in the crowd annotation interface. The grouping was based on the geographical region and the prior land cover class identified at the sample location. |
| PileRegion | Unique number identifying the geographical region (1=Sumatera, 2=Kalimantan, 3=Sulawesi, 4=Papua, 5=Java Madura Bali, 6=Maluku, 7=Nusa Tenggara, 8=Whole Indonesia). |
| PileClass | Unique number identifying the prior land cover class (1=Undisturbed Forest, 2=Logged Over Forest, 3=Oil Palm Monoculture, 4=Tree Based Not Oil Palm, 5=Shrub, 6=Grass and Savanna, 7=Cropland). This corresponds to “SimplifiedClass” field in “landCoverClassesConversion.csv” table. |

**Group 2: CROWD_ANNOTATIONS**

Table S7. Header of crowdAnnotationsRaw.csv.

| **Variable** | **Explanation** |
| --- | --- |
| *NonExpertActivityID* | Sequentially assigned number identifying every submission entered into the mobile application by the crowd/non-expert annotator. |
| NonExpertAnnotation | Unique number identifying the annotation/answer submitted by the crowd/non-expert annotator in the mobile application (1=Yes, 2=No, 3=Maybe). |

Table S8. Header of crowdAnnotationsRawInfo.csv.

| **Variable** | **Explanation** |
| --- | --- |
| *NonExpertActivityID* | Sequentially assigned number identifying every submission entered into the mobile application by the crowd/non-expert annotator. |
| NonExpertActivityTime | Exact time and date when the submission was entered into the mobile application by the crowd/non-expert annotator. |
| SampleID | Unique number identifying each sample pixel (i.e., VHR image chip). |
| NonExpertAnnotatorID | Unique number identifying the crowd/non-expert annotator. |

Table S9. Header of crowdAnnotationsPerAnnotatorMajority_.csv.

| **Variable** | **Explanation** |
| --- | --- |
| *SampleID* | Unique number identifying each sample pixel (i.e., VHR image chip). |
| *NonExpertAnnotatorID* | Unique number identifying the crowd/non-expert annotator. |
| MajorityAnnotationPerSamplePerNonExpert | Unique number identifying the majority annotation/answer submitted by a crowd/non-expert annotator for a sample in the mobile application (1=Yes, 2=No, 3=Maybe). |

Table S10. Head of crowdAnnotationsConsensusPerSample_.csv.

| **Variable** | **Explanation** |
| --- | --- |
| *SampleID* | Unique number identifying each sample pixel (i.e., VHR image chip). |
| NumberOfAnnotationsByNonExpertsAggregated | Number of annotations considered in identifying consensus annotation/answer. |
| ConsensusAnnotationAmongNonExperts | Unique number identifying the consensus annotation/answer among the crowd/non-expert annotators (1=Yes, 2=No, 3=Maybe). |
| ConsensusAnnotationUncertaintyAmongNonExperts | Value of the uncertainty score for the final consensus annotation/answer [fraction]. |

Table S11. Header of crowdAnnotators_expertAgreement_.csv**.**

| **Variable** | **Explanation** |
| --- | --- |
| *NonExpertAnnotatorID* | Unique number identifying the crowd/non-expert annotator. |
| *PileID* | Unique number identifying the pile (i.e., group of items) of which the sample pixel (i.e., VHR image chip) belongs to in the crowd annotation interface. The grouping was based on the geographical region and the prior land cover class identified at the sample location. |
| NumberOfInAppControlItemsAnnotated | Number of unique control items annotated by the crowd/non-expert annotator in that pile. |
| ExpertAgreementExpected | Value of the expected expert agreement of the individual-annotator with the in-app control samples, summarized into per annotator, per image pile [fraction]. |
| ExpertAgreementObserved | Value of the observed expert agreement of the individual-annotator with the in-app control samples, summarized into per annotator, per image pile [fraction]. |
| ExpertAgreementAdjusted_ | Value of the chance-adjusted expert agreement of the individual-annotator with the in-app control samples, summarized into per annotator, per image pile [fraction]. |

Table S12. Header of crowdAnnotators_intraAnnotatorAgreement_.csv.

| **Variable** | **Explanation** |
| --- | --- |
| *NonExpertAnnotatorID* | Unique number identifying the crowd/non-expert annotator. |
| *PileID* | Unique number identifying the pile (i.e., group of items) of which the sample pixel (i.e., VHR image chip) belongs to in the crowd annotation interface. |
| NumberOfRepeatAnnotationsByNonExpert | Total number of repeat annotations (i.e., for the same item) made by that crowd/non-expert annotator in that pile. |
| MedianNumberOfRepeatAnnotationsByNonExpert | Median number of repeat annotations (i.e., for the same item) per item made by that crowd/non-expert annotator in that pile. |
| NumberOfItemsWithRepeatAnnotationsByNonExpert | Number of unique items with repeat annotations by that crowd/non-expert annotator in that pile. |
| IntraAnnotatorAgreeement | Value of the observed intra-annotator agreement of the individual annotator, summarized into per annotator, per image pile [fraction]. |

Table S13. Header of crowdAnnotators_interAnnotatorAgreement_.csv.

| **Variable** | **Explanation** |
| --- | --- |
| *NonExpertAnnotatorID* | Unique number identifying the crowd/non-expert annotator. |
| *PileID* | Unique number identifying the pile (i.e., group of items) of which the sample pixel (i.e., VHR image chip) belongs to in the crowd annotation interface. |
| NumberOfItemsAnnotatedByNonExpert | Number of unique items annotated by that crowd/non-expert annotator in that pile. |
| MedianNumberOfAnnotationsByAllNonExperts | Median number of annotations made by all the crowd/non-expert annotators for the items annotated by that crowd/non-expert annotator in that pile. |
| InterAnnotatorAgreementExpected | Value of the expected inter-annotator agreement of the individual-annotator, summarized into per annotator, per image pile [fraction]. |
| InterAnnotatorAgreementObserved | ­Value of the observed inter-annotator agreement of the individual-annotator, summarized into per annotator, per image pile [fraction]. |
| InterAnnotatorAgreementAdjusted_ | Value of the chance-adjusted inter-annotator agreement of the individual-annotator, summarized into per annotator, per image pile [fraction]. |

Table S14. Header of crowdAnnotatorsSummaryScorePerSamplePerLabel_.csv.

| **Variable** | **Explanation** |
| --- | --- |
| *SampleID* | Unique number identifying each sample pixel (i.e., VHR image chip). |
| NumberOfAllAnnotationsByNonExperts | Number of all annotations made by non expert annotators for the sample. |
| NumberOfYesAnnotationsByNonExperts | Number of “Yes” annotations made by non expert annotators for the sample. |
| NumberOfNoAnnotationsByNonExperts | Number of “No” annotations made by non expert annotators for the sample. |
| NumberOfMaybeAnnotationsByNonExperts | Number of “Maybe” annotations made by non expert annotators for the sample. |
| AverageAnnotatorsScoresYes | The average of the credibility score of the non expert annotators who answered “Yes” for the sample [fraction]. The credibility score is the observed expert agreement based on in-app control samples. |
| AverageAnnotatorsScoresNo | The average of the credibility score of the non expert annotators who answered “No” for the sample [fraction]. |
| AverageAnnotatorsScoresMaybe | The average of the credibility score of the non expert annotators who answered “Maybe” for the sample [fraction]. |
| SumAnnotatorsScoresYes | The sum of the credibility score of the non expert annotators who answered “Yes” for the sample. |
| SumAnnotatorsScoresNo | The sum of the credibility score of the non expert annotators who answered “No” for the sample. |
| SumAnnotatorsScoresMaybe | The sum of the credibility score of the non expert annotators who answered “Maybe” for the sample. |

Table S15. Header of crowdAnnotatorsSummaryScorePerSamplePerLabel_annotatorsFiltered_.csv.

| **Variable** | **Explanation** |
| --- | --- |
| *SampleID* | Unique number identifying each sample pixel (i.e., VHR image chip). |
| NumberOfAllAnnotationsByNonExperts_AnnotatorsFiltered | Number of all annotations made by non expert annotators for the sample, with low-performing annotators excluded. Low-performing annotators are annotators with negative values of chance-adjusted expert agreement score. |
| NumberOfYesAnnotationsByNonExperts_AnnotatorsFiltered | Number of “Yes” annotations made by non expert annotators for the sample, with low-performing annotators excluded. |
| NumberOfNoAnnotationsByNonExperts_AnnotatorsFiltered | Number of “No” annotations made by non expert annotators for the sample, with low-performing annotators excluded. |
| NumberOfMaybeAnnotationsByNonExperts_AnnotatorsFiltered | Number of “Maybe” annotations made by non expert annotators for the sample, with low-performing annotators excluded, with low-performing annotators excluded. |
| AverageAnnotatorsScoresYes_AnnotatorsFiltered | The average of the credibility score of the non expert annotators who answered “Yes” for the sample, with low-performing annotators excluded [fraction]. The credibility score is the observed expert agreement based on in-app control samples. |
| AverageAnnotatorsScoresNo_AnnotatorsFiltered | The average of the credibility score of the non expert annotators who answered “No” for the sample, with low-performing annotators excluded [fraction]. |
| AverageAnnotatorsScoresMaybe_AnnotatorsFiltered | The average of the credibility score of the non expert annotators who answered “Maybe” for the sample, with low-performing annotators excluded [fraction]. |
| SumAnnotatorsScoresYes_AnnotatorsFiltered | The sum of the credibility score of the non expert annotators who answered “Yes” for the sample, with low-performing annotators excluded. |
| SumAnnotatorsScoresNo_AnnotatorsFiltered | The sum of the credibility score of the non expert annotators who answered “No” for the sample, with low-performing annotators excluded. |
| SumAnnotatorsScoresMaybe_AnnotatorsFiltered | The sum of the credibility score of the non expert annotators who answered “Maybe” for the sample, with low-performing annotators excluded. |

**Group 3: EXPERTS_ANNOTATIONS**

Table S16. Header of expertsAnnotationsRaw.csv.

| **Variable** | **Explanation** |
| --- | --- |
| *ExpertActivityID* | Sequentially assigned number identifying every submission entered into the web application by the expert annotator(s). Note the expert annotation was either carried out by a group of experts during workshops, or by an individual expert during the independent individual sessions (see “ExpertInterpretationType” field in “expertsAnnotationsRawInfo.csv” table). |
| ExpertAnnotation | Unique number identifying the label provided by the expert annotator(s) in the expert campaign (1=Undisturbed Dryland Forest, 2=Logged-Over Dryland Forest, 3=Undisturbed Mangrove Forest, 4=Logged-Over Mangrove Forest, 5=Undisturbed Swamp Forest, 6=Logged-Over Swamp Forest, 7=Agroforestry, 8=Plantation Forest, 9=Rubber Monoculture, 10=Oil Palm Monoculture, 11=Other Monoculture, 12=Grass or Savanna, 13=Shrub, 14=Cropland, 15=Settlement, 16=Cleared Land, 17=Waterbody). This corresponds to “DetailedClass” field in “landCoverClassesConversion.csv” table. |
| ExpertConfidenceScoreSelfAssessed | Unique number identifying the self-reported confidence on the annotation submitted (1=low, 2=moderate, 3=high). |
| ExpertCommentTransEng | The comments that the expert annotator entered for some samples, translated to English. |

Table S17. Header of expertsAnnotationsRawInfo.csv.

| **Variable** | **Explanation** |
| --- | --- |
| *ExpertActivityID* | Sequentially assigned number identifying every submission entered into the web application by the expert annotator. Note the expert annotation was either carried out by a group of experts during workshops, or by an individual expert during the independent individual sessions (see “ExpertInterpretationType” field). |
| ExpertActivityTime | Exact time and date when the submission was entered into the system by the expert annotator(s). |
| ExpertAnnotatorID | Unique number identifying the expert annotator(s) in the web application. |
| SampleID | Unique number identifying each sample pixel (i.e., VHR image chip). |
| ExpertAnnotationSampleGroup | Unique number identifying the sample group and thus interpreter group during the expert workshops (1=Sumatera, 2=Java Madura Bali, 3=Kalimantan, 4=Sulawesi Maluku, 5=Papua Nusa Tenggara, 6=Whole Indonesia i.e., samples not divided into groups in the annotation interface). |
| ExpertInterpretationType | Unique number identifying whether the expert annotation was provided during group session or independent individual session (1=group session, 2=individual session). |

Table S18. Header of expertsCommentsTrans.csv.

| **Variable** | **Explanation** |
| --- | --- |
| *ExpertCommentTransEng* | The English translation of the comments that the expert annotator entered for some samples. |
| ExpertCommentOrigInd | The original comments that the expert annotator entered for some samples. |

Table S19. Header of expertsAnnotationsConsensus_.csv.

| **Variable** | **Explanation** |
| --- | --- |
| *SampleID* | Unique number identifying each sample pixel (i.e., VHR image chip). |
| ConsensusAnnotationAmongExperts | Unique number identifying the consensus label among the expert annotators in the expert campaign (1=Undisturbed Dryland Forest, 2=Logged-Over Dryland Forest, 3=Undisturbed Mangrove Forest, 4=Logged-Over Mangrove Forest, 5=Undisturbed Swamp Forest, 6=Logged-Over Swamp Forest, 7=Agroforestry, 8=Plantation Forest, 9=Rubber Monoculture, 10=Oil Palm Monoculture, 11=Other Monoculture, 12=Grass or Savanna, 13=Shrub, 14=Cropland, 15=Settlement, 16=Cleared Land, 17=Waterbody). This corresponds to “DetailedClass” field in “landCoverClassesConversion.csv” table. |
| ConsensusTypeAmongExperts | Unique number identifying the form of consensus reached among the experts for a given sample (1=both experts agree, 2=two experts agree out of three experts, 3=all three experts agree, 4=three experts agree out of four experts, 5=all four experts agree, 6=group session). |

**Group 4: CROWD_CONSENSUS_VS_EXPERTS_CONSENSUS_ANNOTATIONS**

Table S20. Header of crowdConsensusVsExpertsConsensusAnnotations_.csv.

| **Variable** | **Explanation** |
| --- | --- |
| *SampleID* | Unique number identifying each sample pixel (i.e., VHR image chip). |
| ConsensusAnnotationAmongNonExperts | Unique number identifying the consensus annotation/answer among the crowd/non-expert annotators in the crowdsourcing campaign (1=Yes, 2=No, 3=Maybe). |
| ConsensusAnnotationAmongExpertsBinarized | Unique number identifying the consensus annotation/answer among the expert annotators in the expert campaign (1=Yes, 2=No, 3=Maybe). |

**Group 5: AUXILIARY_FILES**

Table S21. Header of landCoverClassesConversion.csv.

| **Variable** | **Explanation** |
| --- | --- |
| *DetailedClass* | Unique number identifying the detailed land cover class used in the expert annotation process (1=Undisturbed Dryland Forest, 2=Logged-Over Dryland Forest, 3=Undisturbed Mangrove Forest, 4=Logged-Over Mangrove Forest, 5=Undisturbed Swamp Forest, 6=Logged-Over Swamp Forest, 7=Agroforestry, 8=Plantation Forest, 9=Rubber Monoculture, 10=Oil Palm Monoculture, 11=Other Monoculture, 12=Grass or Savanna, 13=Shrub, 14=Cropland, 15=Settlement, 16=Cleared Land, 17=Waterbody). See long description of the definition of the classes in “landCoverClassesDefinition.docx” file. |
| SimplifiedClass | Unique number identifying the simplified land cover class used in the non-expert (crowdsourced) annotation process (1=Undisturbed Forest, 2=Logged Over Forest, 3=Oil Palm Monoculture, 4=Tree Based Not Oil Palm, 5=Shrub, 6=Grass and Savanna, 7=Cropland, 8=Does Not Belong To Any Simplified Class). This corresponds to “PileClass” field in “piles.csv” table. |

Table S22. Definition of the land cover classes as provided by the local experts.

| **Class ID (“Detailed**  **Class”)** | **Class name (Indonesian)** | **Class name (translated)** | **Class definition (Indonesian)** | **Class definition (translated)** |
| --- | --- | --- | --- | --- |
| 1 | “Hutan lahan kering primer” | Undisturbed Dryland Forest | “Tutupan hutan alami dengan kanopi yang rapat (>80%), spesies yang sangat beragam dan basal area yang relative tinggi. Secara mudah, hutan ini diindikasikan tidak adanya jalan logging. Pada citra satelit, diindikasikan dengan nilai index vegetasi dan band infrared yang tinggi, dan band tampak (visible) yang rendah.” | “Natural forest cover with dense canopy (>80%), very diverse species, and relatively high basal area. They are indicated by the absence of logging roads. In the satellite image, they are indicated by high values of vegetation index and infrared band reflectance, and low values of visible bands reflectance.” |
| 2 | “Hutan lahan kering sekunder” | Logged-Over Dryland Forest | “Tutupan hutan alam dengan kerapatan pohon yang bervariasi (30% - 80%) yang telah mengalami gangguan aktivitas manusia maupun aktivitas alam lainnya yang biasanya dicirikan oleh adanya jalan logging maupun bekas tebangan.” | “Natural forest cover with varying tree density (30% - 80%) that has experienced disturbances from human activities or other natural activities, usually characterized by the presence of logging roads or logging signs/marks.” |
| 3 | “Hutan mangrove primer” | Undisturbed Mangrove Forest | “Tutupan hutan yang didominasi oleh pohon bakau yang berlokasi pada pesisir pantai dan tidak pernah mengalami penebangan maupun aktivitas manusia lainnya.” | “Forest cover dominated by mangrove (‘bakau’) trees, located on the coast, and has never experienced logging or other human activities “ |
| 4 | “Hutan mangrove sekunder” | Logged-Over Mangrove Forest | “Tutupan hutan yang didominasi oleh pohon bakau yang telah mengalami degradasi berlokasi di sekitar pesisir pantai dan pernah mengalami penebangan maupun aktivitas manusia lainnya.” | “Forest cover dominated by mangrove (‘bakau’) trees that has experienced degradation, located around the coast, and has experienced logging or other human activities.” |
| 5 | “Hutan rawa primer” | Undisturbed Swamp Forest | “Tutupan vegetasi alami yang berada pada lahan basah yang tergenang sementara maupun permanen, tidak pernah mengalami penebangan masalalu ataupun pengaruh aktivitas manusia yang biasanya dicirikan dengan mudah tidak adanya jalan logging, parit, maupun kanal.” | “Natural vegetation cover that is situated on wetlands that are temporarily or permanently inundated, that has never experienced past logging or impacts of human activities that is usually characterized by the absence of logging roads, ditches, or canals.” |
| 6 | “Hutan rawa sekunder” | Logged-Over Swamp Forest | “Tutupan vegetasi alami yang berada pada lahan basah yang tergenang sementara maupun permanen, yang telah mengalami penebangan masalalu ataupun pengaruh aktivitas manusia yang biasanya dicirikan dengan mudah dengan adanya jalan logging, parit, maupun kanal.” | “Natural vegetation cover that is situated on wetlands that are temporarily or permanently inundated, that has experienced past logging or impacts of human activities that is usually characterized by the presence of logging roads, ditches, or canals.” |
| 7 | “Agroforestri” | Agroforestry | “Tutupan vegetasi yang terdiri dari campuran komoditas perkebunan, buah-buahan, pohon berkayu, maupun tanaman semusim lainnya yang tumbuh secara bersama-sama dalalam satu waktu atau rotasi di dalam satu lahan.” | “Vegetation cover consisting of a mixture of estate/plantation commodities, fruits, woody trees, or other seasonal plants/crops that grow together at one time or rotation within one land area/field.” |
| 8 | “Hutan tanaman” | Plantation Forest | “Tutupan vegetasi pohon berkayu baik kayu lunak maupun keras yang ditanam secara monokultur untuk tujuan komersial. Hutan tanaman dalam skala luas biasanya dijalankan oleh pemegang konsesi, sedangkan dalam skala kecil biasanya dikelola oleh masyarakat local. Komoditasnya biasanya akasia, eucalyptus, pinus, jati, sengon, damar, dan lain-lain.” | “Vegetation cover of woody trees, both softwood and hardwood, which are planted in monoculture for commercial purpose. Plantation Forest in a large scale are usually run by concession holders, whereas in a small scale usually managed by local communities. The commodities are usually acacia, eucalyptus, pine, teak, sengon, resin, and others.” |
| 9 | “Karet monokultur” | Rubber Monoculture | “Tutupan vegetasi yang didominasi hampir 100% oleh tanaman karet dalam satu lahan.” | “Vegetation cover that is dominated almost 100% by rubber trees within one area/field.” |
| 10 | “Kelapa sawit monokultur” | Oil Palm Monoculture | “Tutupan vegetasi yang didominasi hampir 100% oleh tanaman kelapa sawit dalam satu lahan.” | “Vegetation cover that is dominated almost 100% by oil palm trees within one land area/field.” |
| 11 | “Monokultur lain” | Other Monoculture | “Tutupan vegetasi yang didominasi hampir 100% oleh tanaman perkebunan lain selain karet dan kelapa sawit dalam satu lahan.” | “Vegetation cover that is dominated almost 100% by monoculture/plantation crops, other than rubber or oil palm, within one land area/field.” |
| 12 | “Rerumputan/padang rumput/savanna” | Grass or Savanna | “Tutupan vegetasi yang didominasi oleh tutupan rumput yang tumbuh secara alami. | “Vegetation cover dominated by grass cover that grows naturally.” |
| 13 | “Semak belukar” | Shrub | “Tutupan vegetasi yang didominasi oleh vegetasi bukan pohon dengan ketinggian tidak lebih dari 5-6 m, biasanya hasil dari perladangan berpindah, atau bekas tebangan yang telah terdegradasi, maupun suksesi alami dengan tegakan pohon yang belum terbentuk yang berumur 2-3 tahun.” | “Vegetation cover dominated by non-tree vegetation with a height of not more than 5-6 m, usually the result of shifting cultivation, or ex logging areas that have been degraded, or natural succession with yet-to-form tree stands that are 2-3 years old.” |
| 14 | “Lahan pertanian” | Cropland | “Tutupan vegetasi yang didominasi oleh tanaman padi (sawah), atau tanaman palawija maupun hortikultura (pertanian lahan kering).” | “Vegetation cover dominated by rice (paddy), or “palawija” or horticultural plants/crops (dryland agriculture)”.  “Palawija” crops: secondary food crops i.e. usually grown after main crop i.e. rice, on dryland. Examples are groundnuts, maize, cassava, soybean, and roots/pulses. Horticultural crops: fruits, vegetables, herbs, medicinal and ornamental plants. |
| 15 | “Permukiman” | Settlement | “Tutupan yang didominasi oleh bangunan.” | “Surface cover dominated by buildings.” |
| 16 | “Lahan terbuka” | Cleared Land | “Tutupan lahan yang hampir 100% tanpa tutupan vegetasi.” | “Land cover that is almost 100% without vegetation cover.” |
| 17 | “Tubuh air” | Waterbody | “Tutupan yang didominasi oleh air, biasanya direpresentasikan dengan sungai, danau, waduk, tambak, dll.” | “Surface cover dominated by water, usually represented as rivers, lakes, reservoirs, ponds, etc.” |
